# OA14.02. Exploration of body awareness and pain and emotion regulation among yoga and meditation practitioners: does type of mind-body practice matter?

**DOI:** 10.1186/1472-6882-12-S1-O54

**Published:** 2012-06-12

**Authors:** J Daubenmier, W Mehling, C Price, E Bartmess-Levasseur, M Acree, A Stewart

**Affiliations:** 1 University of California, San Francisco, San Francisco, USA; 2 University of Washington, Seattle, USA

## Purpose

Yoga and meditation are mind-body therapies that are effective for managing pain and negative emotions. Body awareness is a key component of these therapies and may be an important mechanism of action. It is unknown whether differences exist between yoga and meditation practitioners in body awareness and pain and emotion regulation. We explored these questions: do yoga and meditation practitioners differ in degree of body awareness and ability to regulate pain and negative affect? Is body awareness more strongly related to pain and emotional regulation outcomes among yoga or meditation practitioners?

## Methods

We used a convenience sample of yoga (N=88) and meditation practitioners (N=112) participating in a study to develop the Multi-Dimensional Assessment of Interoceptive Awareness Instrument (MAIA). We compared mean scores of yoga practitioners and meditators along eight dimensions of body awareness using the MAIA: Noticing, Distracting, Worrying, Attention Regulation, Emotional Awareness, Self-Regulation, Body Listening, and Trusting, and measures of pain and emotion regulation: Pain Catastrophizing Scale (3 subscales), Anxiety Sensitivity Inventory (2 subscales), and Difficulties in Emotion Regulation Scale (6 subscales). Within each group, we examined correlations between MAIA subscales and pain and emotional regulation measures.

## Results

Yoga practitioners reported significantly higher levels of Noticing, Emotional Awareness, Trust, and more Worrying. Yoga practitioners reported significantly higher levels of Noticing, Emotional Awareness, Trust, and more Worrying. Groups did not differ in pain or emotion regulation. In each group, greater body awareness was significantly associated with less pain catastrophizing, less fear of symptoms associated with arousal, and less difficulty managing negative emotions. The pattern of correlations between MAIA subscales and pain and emotion regulation subscales did not differ substantially between meditation and yoga practitioners.

## Conclusion

Although meditators reported lower levels of specific aspects of body awareness, body awareness cultivated in either meditation or yoga may improve pain management and emotion regulation.

